# The sexual function of women with epilepsy: a comparative study

**DOI:** 10.1192/j.eurpsy.2022.721

**Published:** 2022-09-01

**Authors:** N. Sayari, A. Maamri, O. Charaa, A. Tajmout, A. Hajri, H. Zalila

**Affiliations:** Razi Hospital, Emergency And Outpatient Departement, manouba, Tunisia

**Keywords:** sexual function, sexuality, epilepsy, neurology

## Abstract

**Introduction:**

Epilepsy is a neurological disease that interferes negatively with many areas of the patient’s life. Sexual dysfunction is a frequent comorbidity in epileptic patients. Quality of life is particularly affected in women, who are also culturally stigmatized because of their illness.

**Objectives:**

To assess the sexual function of women with epilepsy compared to healthy women.

**Methods:**

This was a case-control study of female patients with generalized epilepsy, carried out at the national institute of neurology in Tunisia in 2018. The controls were the patients’ companions in the department. They were matched by age with the cases. Sexual function was assessed by the female sexual function index (FSFI).

**Results:**

We included 40 cases and 40 controls.Their averge age was 30.45years. In comparison to the controls,the women with epilepsy had less kids ( p=0.04) and more miscarriages (p=0.032). On the other hand, women with epilepsy presented more sexual dysfunctions (p=0. 03) and had a lower total score on the FSFI (p=0.015) as well as significantly lower scores in the domains “desire” (p=0.009), “orgasm” (p=0.026), “satisfaction” (p=0.001) and pain (p=0.015).

**Conclusions:**

The findings of this study are consisting with the literature, the women with epilepsy in this survey had impaired sexual function. More attention should be paid to these sexual disorders, previously considered secondary or even neglected.

**Disclosure:**

No significant relationships.

